# Long-term outcomes of limb salvage treatment with custom-made extendible endoprosthesis for bone sarcoma around the knee in children

**DOI:** 10.1186/s13018-019-1534-x

**Published:** 2020-01-16

**Authors:** Changye Zou, Zhiqiang Zhao, Tiao Lin, Yongfu Huang, Xianbiao Xie, Junqiang Yin, Gang Huang, Bo Wang, Jingnan Shen

**Affiliations:** 1 Department of Musculoskeletal Oncology, The First Affiliated Hospital of Sun Yat-Sen University, 58#, Zhongshan 2 Road, Guangzhou, 510080 China; 2 Guangdong Provincial Key Laboratory of Orthopedics and Traumatology, The First Affiliated Hospital of Sun Yat-sen University, 58#, Zhongshan 2 Road, Guangzhou, 510080 China; 3 Department of Orthopedic, The First Affiliated Hospital of Sun Yat-Sen University, 58#, Zhongshan 2 Road, Guangzhou, 510080 China

**Keywords:** Osteogenic sarcoma, Prosthesis survival, Limb length discrepancy, Aseptic loosening

## Abstract

**Background:**

Limb salvage for bone sarcoma around the knee in skeletally immature children is challenging because of interference on two critical growth plates in the lower limb. This retrospective study aims to evaluate long-term outcomes and influence on growth of the lower limb of the cemented extendible endoprostheses.

**Methods:**

Forty-five children with bone sarcoma around the knee, who underwent custom-made extendible endoprosthesis replacements, were included in this study. The average follow-up was 10.1 years. Survival, prosthetic-related complications and revision, functional outcomes, and influence on growth by prosthesis implantation were recorded.

**Results:**

The 5-year disease-free survival and overall survival are 54.9% and 72.7%, and the 5-year prosthesis survival rate is 59.4%. The prosthesis was extended 4.2 cm in average. Limb length discrepancies of 20 patients were within 2 cm, and growth inhibition of proximal tibial epiphysis by passive implant insertion was observed. Aseptic loosening in 7 patients was the most significant complication. The Musculoskeletal Tumor Society score at last visit was 83.2%.

**Conclusions:**

The use of custom-made extendible endoprosthesis provided good functional results for children with bone tumor around the knee. Further improvement of the prosthesis design and operation technique will help to decrease complication and gain better limb function.

## Introduction

Limb salvage has been the first choice for the treatment of primary malignant bone tumor of lower extremity in 80% of patients with the advance in the diagnostic imaging, chemotherapy, biomedical engineering, and surgical technique [1]. Bone sarcoma frequently involves the metaphysis around the knee, and the removal of the growth plate during wide resection of the tumor can result in significant limb length discrepancies (LLDs) at skeletally immature children. The reconstruction with endoprosthesis implant mostly will interfere the adjacent normal growth plate of the same joint, and the LLD thus would emerge unavoidably as the contralateral extremity continues to grow normally. An increasing requirement of limb length equality not only preserves the limb but also maximizes its function and minimizes complications after resection of the growth plates.

The extendible endoprosthesis replacement has been in use since 1976 [2]. With the development of prosthesis design, limb lengthens procedure experienced from fully exposed surgery to minimally invasive surgery. The noninvasive expansion mechanism with a rotating external magnet or an electromagnetic field has also been adopted [3]. The medium-term result showed that the noninvasive extendible endoprostheses in pediatric bone tumor patients achieve the goal of atraumatic and accurate lengthening with reduced mid-term morbidity and excellent functional outcome, even though complications such as high rate of infections and failure of lengthening mechanism could not be avoided [4]. While before the noninvasive extendible was available worldwide, the domestic extendible prosthesis has been playing a critical role to salvage the limb of pediatrics patients with bone tumor in China, which could be extended by a minimal incision.

From 2002, we had applied the custom-made extendible prosthesis for adolescent patients younger than 14 years old with sarcoma in the lower limb. In the current study, we aim to assess the long-term result, influence of prosthesis on the adjacent normal growth plate, complications such as infection, mechanical failure, fractures, neurovascular damage, and collapse of prosthesis. We also propose certain recommendations to address the complications.

## Materials and methods

### Patient population

The clinical data for patients undergoing limb salvage with extendible prosthesis from 2002 to 2011 were retrieved from our department retrospectively, with ethics approval from our institutional ethics committee. The inclusion criteria for this study are as follows: (1) Patients who suffered from sarcoma around the knee joint were less than 14 years old, and the expected limb growth was more than 4 cm according to the multiplier method of Paley et al. [5]. (2) Tumor could be resected with safe margins, and the epiphysis of the tumor-affected bone could not be conserved. (3) Crucial nerve and vessels such as tibial nerve and popliteal vessels were not affected by the tumor. (4) Patients were not categorized as Enneking stage III. (5) Karnofsky performance score (KPS) > 60.

Clinical data such as age, tumor location, histological result, operation date, and times and length of prosthesis lengthening were recorded. Forty-five patients (26 boys and 19 girls) were included (Table 1). Primary diagnoses were osteosarcoma in 44 patients and Ewing’s sarcoma in 1 patient. The average age at diagnosis was 10.1 years (range from 6 to 14). Thirty-five patients had the sarcoma in the distal femur and 10 patients in the proximal tibia.

**Table 1:** summary data of the patients

	General information		Number	Percentage
For all patients (45)	Age (years)	≦10	23	51.1%
		>10	22	48.9%
	Gender	Female	19	42.2%
		Male	26	57.8%
	Location	Femur	35	77.8%
		Tibia	10	22.2%
	Tumor size	≦8cm	10	22.2
		>8cm	35	77.8
	Pathology	Osteosarcoma	44	97.8%
		Ewing sarcoma	1	2.2%
	Metastasis	Yes	15	33.3%
		No	30	66.7%
	Survival	DOD	12	26.7%
		AWD	3	6.7%
		AW/OD	30	66.7%
For survived patients (33)	Limb salvage	Yes	31	93.9%
		No	2	6.1%
	Prosthesis survival	Yes	21	63.6%
		No	12	36.4%
	prosthesis extension	Yes	22	66.70%
		No	11	33.3%
	LLD	≦2cm	20	60.6%
		>2cm	13	39.4%

*DOD* dead of disease, *AWD* alive with disease, *AW/OD* alive without disease

### Extendible prosthesis

**Fig. 1:**
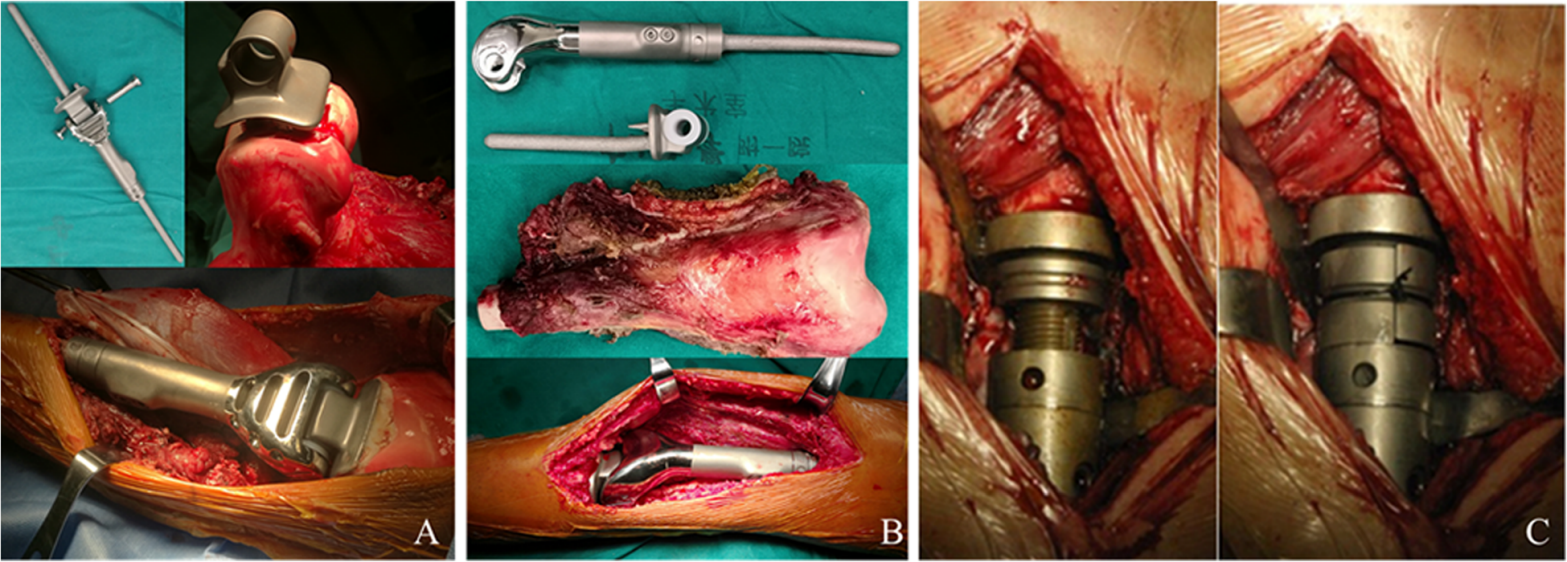
Diagram showing the structure and extension mechanism of the prothesis for patients with the lower limb osteosarcoma. **a** The prothesis used in patients with osteosarcoma in proximal tibia; only partial cartilage and cortex of the distal femur were resected to implant the femoral joint surface and shaft. **b** The prothesis used in patients with osteosarcoma in distal femur. **c** The prosthesis was lengthened by rotating the ring, and the extension of the prosthesis was further secured by inserting two hemicyclic collars between the extension space

All the extendible prostheses used in this study were individually designed by the surgeon in charge and produced by the LDK® company (Beijing, China). The prosthesis consists of a femoral component, a constrained articular component, and a tibial component. The lengthening part composed of a hollow shaft and a piston with screws, which could be lengthened by rotating the ring after removing the locking nail. The extension of the prosthesis was further secured by inserting two hemicyclic collars between the piston and shaft and locked by fastening the nail (Fig. 1). To decrease the interference on growth of adjacent epiphysis and preserve bone mass, the non-lengthening component was designed with a thin stem (diameter less than 10 mm) and a surface with two short anchor nails to prevent rotating.

### Chemotherapy and surgeries

The diagnosis of all the patients were made by a multidisciplinary team based on the symptom and signs, radiology examination, and pathological results of core needle or open biopsy. After diagnosis, the patients were staged using X-ray and MR for the local affected limb, and pulmonary CT scan and bone scan for potential metastasis according to the Enneking staging system. One cycle of neoadjuvant chemotherapy including HDMTX-CF (10–12 g/m^2^), adriamycin (ADM, 50 mg/m^2^) + cisplatin (DDP, 100 mg/m^2^), and ifosfamide (IFO, 12.5 mg/m^2^) was prescribed for osteosarcoma and six cycles of AVI ((ADM, 50 mg/m^2^; VP-16, 100 mg/m^2^; IFO, 12.5 mg/m^2^) for Ewing’s sarcoma. The operation was performed 2 weeks after the chemotherapy for the patients’ recovery.

Before operation, a wide surgical margin of resection was determined according to the MRI results, which was 2 cm away from the reactive zone of the tumor. If the crucial vessels were in the reactive zone but outside of the tumor, the vascular sheath would be separated and resected. An extensive en bloc resection of the tumor including the biopsy tunnel was performed by one group of musculoskeletal oncological surgeons. Cement was used to fix the stem of the extendible component. For the adjacent normal bone of the joint, the cartilage surface was removed with the epiphysis preserved. In all cases of sarcoma located in the proximal tibia, patellar tendon was fixed to the tibial component of the prosthesis and gastrocnemius flap reconstruction was undertaken to cover the prosthesis and suture with the patellar tendon for reconstructing the extensor mechanism. Then, the affected limb was fixed in extension position with cast for 6 weeks for the biological fusion of patellar tendon. The rehabilitation of knee joint was initiated immediately after operation for patients with sarcoma in femur, while it will be delayed for 6 weeks for patients with sarcoma in tibia until the cast was removed.

### Lengthening and follow-up

After the treatment was completed, the patients were surveyed every 3 months for 2 years, every 4 months for year 3, then every 6 months for years 4 and 5, and annually thereafter. Surveillance includes complete physical examination, long leg X-ray of both sites, CT scan for chest, and bone scan if bone metastasis was suspected. The functional performance was evaluated according to the Musculoskeletal Tumor Society (MSTS) Functional Scoring System at the last follow-up [6]. Prosthetic survival was defined as the time between the implantation to removal/revision. Lengthening procedure was started after the completion of chemotherapy and when the LLD is more than 2 cm according to the full leg X-ray film. Extending ring was exposed by a small incision, and 1 to 2 cm was gained in each operation.

**Fig. 2:**
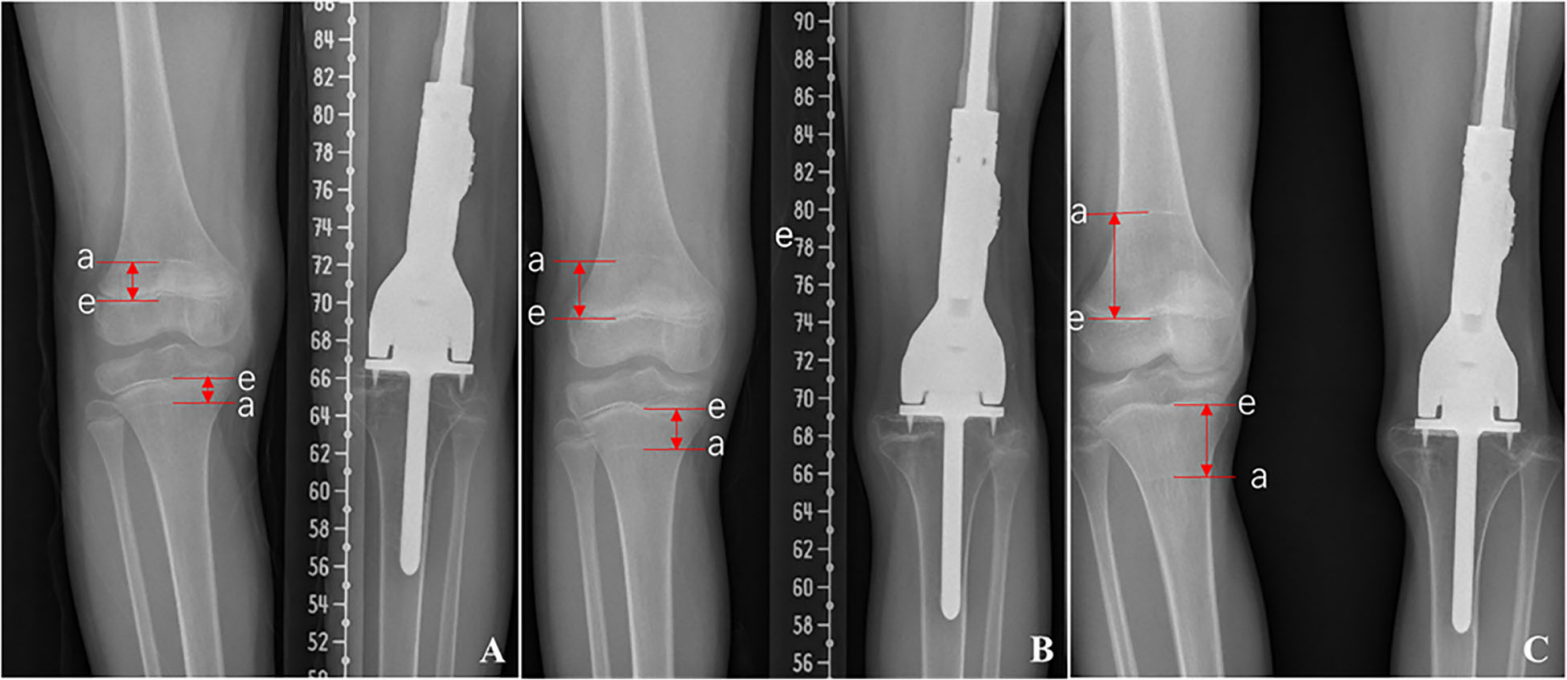
Diagram showing the growth of epiphysis of contralateral healthy proximal tibia and distal femur at different time points after operation. **A** 12 months. **B** 24 months. **C** 48 months in a male patient. a, arrest line; e, epiphysis line. The distance between a and e lines is the growth of the epiphysis after operation

To evaluate the growth of the prosthesis-implanted bone and the contralateral healthy bone, arrest line was used as the starting point for bone growth after the operation. Arrest line is a marker of growth arrest caused by chemotherapy [7]. The growth of each epiphysis was measured by the length between the arrest line and new epiphysis lines (Fig. 2). The length of tibia and the growth of proximal and distal epiphysis of the tibia were measured in the X-ray film periodically. The contribution of both proximal and distal epiphysis to the length of tibia was evaluated. The length of both tibias was compared, and the differences of growth potential of both proximal and distal epiphysis of tibia were compared.

### Statistical methods

All statistical analyses were performed with SPSS17.0 software (IBM Corporation, Somers, NY, USA). Measurements were analyzed by two-tailed Student’s *t* test or one-way analysis of variance. The Kaplan-Meier method was used to calculate the overall survival, disease-free survival, and prosthetic survival. Differences were determined by the log-rank test. *P* < 0.05 was considered statistically significant. Data are shown as mean ± SD.

## Results

### Oncological result

The mean follow-up is 83 months (range from 2 to 180 months) for all the patients and 101.5 months (range from 47 to 180 months) for the surviving patients. R0 resection was achieved in all patients according to postoperative pathology. Fifteen patients developed distant metastasis including 12 patients with lung metastasis, 1 patient with bone metastasis, and 2 patients with lung and bone metastasis. Three patients had local recurrence, which lead to 6.7% of local recurrence rate. Twelve patients died of tumor distant dissemination, and 3 patients live with stable disease. The 5-year disease-free survival and overall survival are 54.9% and 72.7%, respectively (Fig. 2A, B).

### Prosthesis survival

**Fig. 3:**
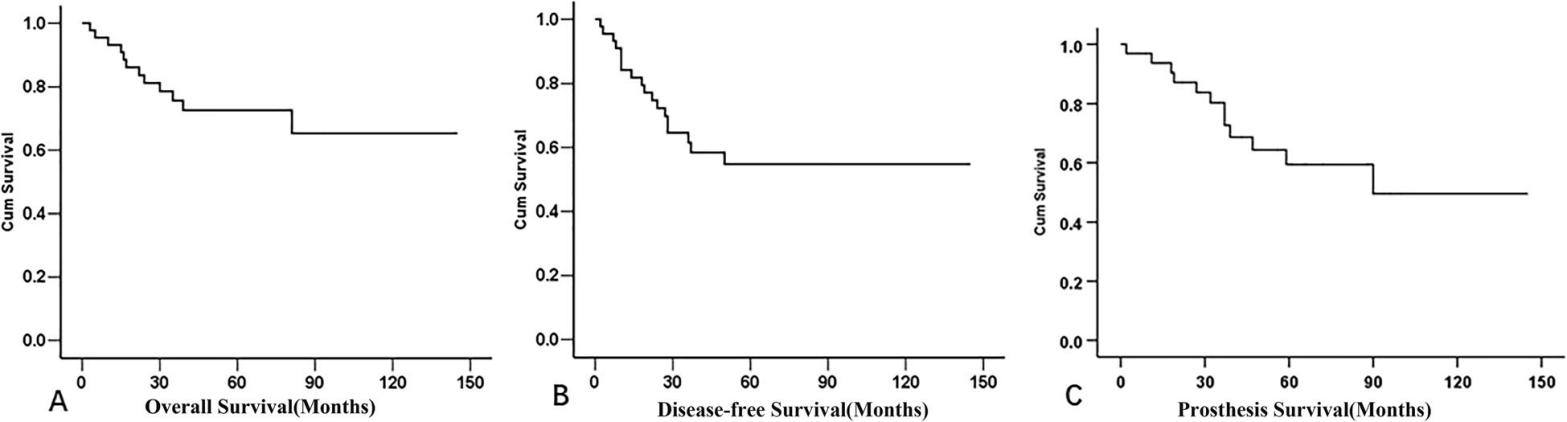
The Kaplan-Meier curve of the overall survival (**a**), the disease-free survival (**b**), and the prosthesis survival (**c**) of the patients

The patients who died with their original prostheses were censored. Among the 33 survivors, 2 patients had amputation because of infection in 1 case and local recurrence in another case. Sixteen revision operations were carried out in 12 patients for infection in 4 cases including 1 patient with additional tear of patellar tendon, aseptic loosening in 7 cases, periprosthetic fracture in 1 case, and twice dislocation of endoprosthesis in 1 case. Ten patients had the prosthesis revision. Using revision or removal of the stemmed components for any reason as an endpoint, the 5-year prosthesis survival rate is 59.4% (Fig. 3c).

### Elongation and limb length

Seventy-seven elongation procedures were carried out for 22 survived patients, 3.5 times (range from 1 to 7) for each patient on average. One to 2 cm was increased each time according to the tension of soft tissue, 1.2 cm (from 1to 2 cm) on average. The prosthesis was extended 4.2 cm on average (from 2 to 7 cm). The LLD of 20 patients was within 2 cm, 1 patient had 6 elongation procedures before out of length of the prosthesis and refused to replace prosthesis, and LLD is 3 cm. Another patient had prosthesis loosening and subsidence, and the LLD was 9 cm before revision. Traction could not restore the limb length completely, and LLD is 5 cm after elongation.

Four patients did not lengthen the prosthesis because LLD is less than 2 cm until bone maturation; all of them were girls of 12 years old when the operation was performed. The other six patients did not extend the prosthesis as planned because of their persistent diseases and individual reasons, and the LLD range from 3 to 8 cm in this group.

**Fig. 4:**
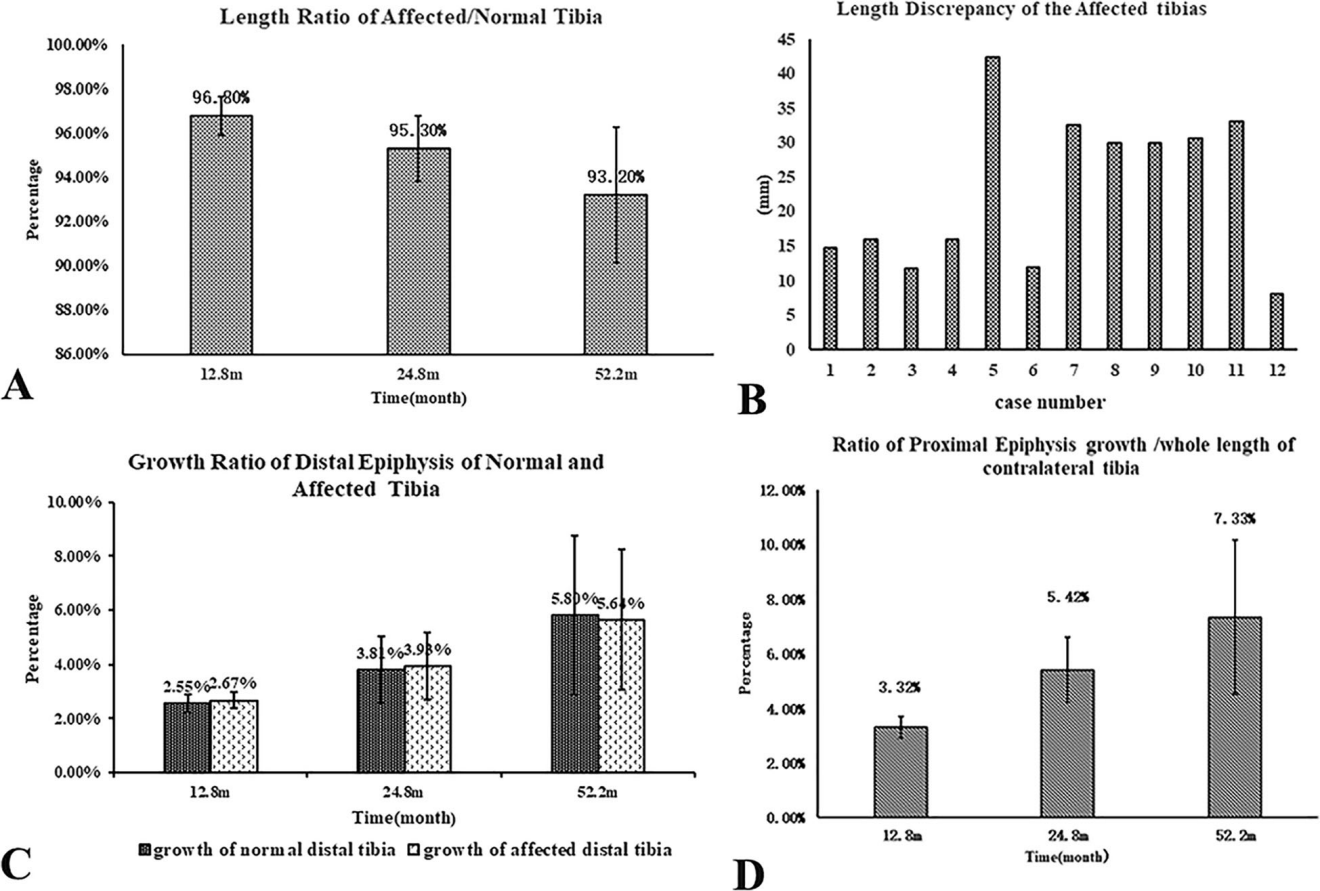
The influence of the passive implant insertion on the growth of the affected tibia compared to the contralateral normal one. **a** The mean length ratio of the affected tibia/control normal tibia. **b** The length discrepancies of the affected tibia compared to the normal one. **c** The ratio of the growth of both distal tibia epiphysis to the whole length of normal tibia. **d** The ratio of the growth of normal proximal tibia epiphysis to the whole length of normal tibia

The LLD is mainly caused by resection of tumor involving epiphysis, while interference of the adjacent normal epiphysis when the extendible endoprosthesis was implanted can never be ignored. As most of the patients in our study had sarcoma in the distant femur, we evaluate to what extent the growth potential of the proximal epiphysis of tibia was inhibited after passive implant insertion comparing with the normal limb. A total of 12 patients with the data required were included in this study. The age was 9.75 ± 1.48 (range from 6 to 12) years old, and the mean follow-up is 6.3 ± 2.1 years. The mean length ratio of the affected tibia/control normal tibia was 96.83% ± 0.87% (range from 95.65 to 97.80%) 12.8 months after operation, 95.30% ± 1.47% (range from 92.38 to 96.80%) 24.8 months after operation, and 93.33% ± 3.11% (range from 88.50 to 95.88%) 52.2 months after operation (Fig. 4a). The average LLD between two tibias was 23.1 mm (range from 8 to 42.4 mm) (Fig. 4b). The contribution of distal epiphysis was 2.59% ± 0.53%, 3.81% ± 1.23%, and 5.78% ± 2.94% at the normal side and 2.67% ± 0.31%, 3.93% ± 1.23%, and 5.64% ± 2.58% at the affected side accordingly (Fig. 4c). There is no significant difference between the growth of the normal and affected distal epiphysis (*P* > 0.05). All the proximal arrest line at the uninterrupted limb was clear to measure. The contribution of the proximal epiphysis to the full length of tibia in the normal limb was 3.23% ± 0.39%, 4.96% ± 1.19%, and 6.84% ± 2.69% accordingly (Fig. 4d). In most cases, the arrest line of the affected proximal tibia was obscure; thus, the growth of the proximal epiphysis was difficult to measure. Since the growth of distal tibia epiphysis in the affected and normal limb was nearly equal, it indicated that the length discrepancy in the tibia was owning to the growth inhibition of proximal epiphysis by the passive insertion of the implant.

### Complication

**Fig. 5:**
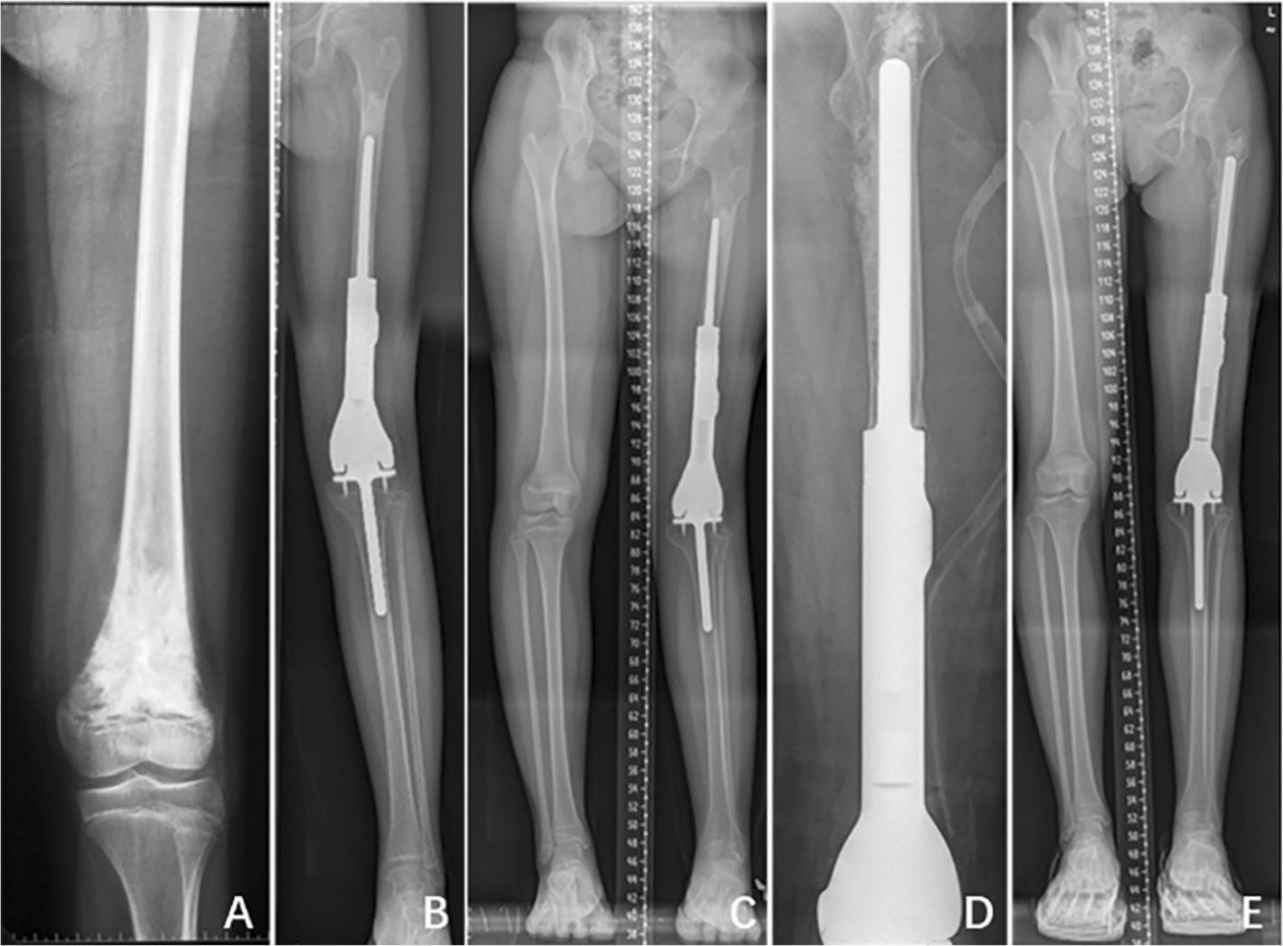
The lengthening and revision of a 9-year-old girl with osteosarcoma in the left distal femur. **a** X-ray indicates osteosarcoma in the left distal femur. **b** The patient underwent tumor resection and implantation of extendible prosthesis. **c** Prosthesis displacement and out of length after extension of 4 cm in 2 years. **d** The revision operation was performed, and a new prosthesis with longer and thicker stem was implanted. **f** The LLDs were less than 1 cm after another 2 cm extension

**Fig. 6:**
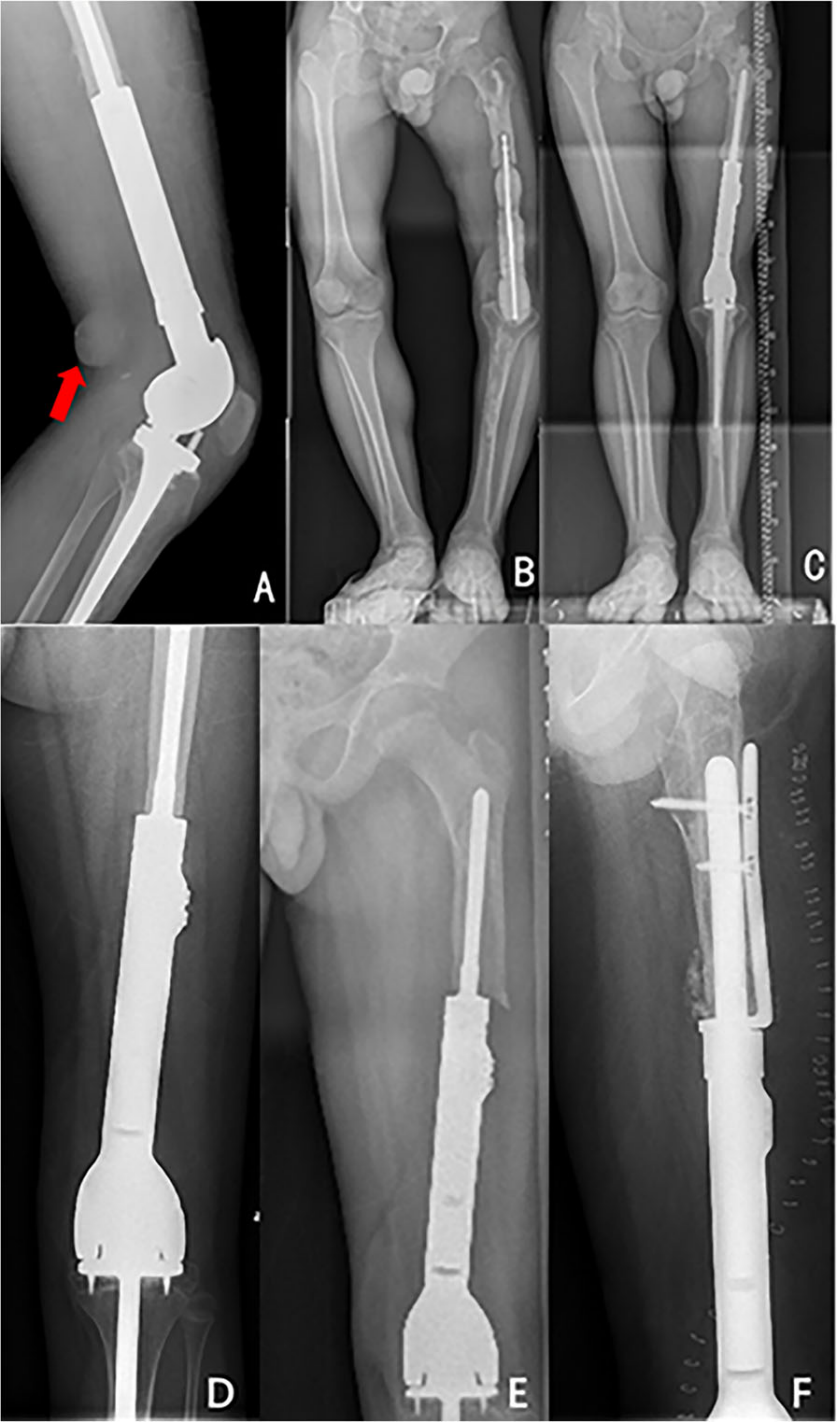
Revision of a patient with periprosthesis infection (**a**–**c**) and a patient with loosening of prosthesis (**d**–**f**). An abscess was observed 4 months after the operation as indicated by the arrow (**a**). Surgical debridement was performed to remove the infected prosthesis, and bone cement spacer containing antibiotics was implanted (**b**). The bone cement spacer was replaced by the prosthesis when there were no signs of infection (**c**). An extension prosthesis was applied for a boy with osteosarcoma in the left femur (**d**). The prosthesis was loosening 1 year after the operation (**e**). A new prosthesis with plate and screw was used to replace the loose one (**f**)

The postoperation complications were observed in 16 patients, including aseptic loosening, deep infection, periprosthesis fracture and dislocation, tear of patellar tendon, and injury of nervous peroneus communis. Aseptic loosening occurred in 7 patients, which was the most significant complication especially in younger patients. The average age of patients with aseptic loosening is 9 years old (range from 6 to 11) at operation, which occurs from 14.7 to 35.4 months after prosthesis implantation (mean of 25.5 months). All aseptic loosening occurred in femur, and none of them was observed in the stem of tibia. Four patients accepted revision operation with newly designed custom-made prosthesis, in which a longer and thicker stem of femur component was applied (Fig. 5), while three of them have plate and screws to stabilize the prosthesis because of the short remnant bone of the femur (Fig. 6e, f). The previous prosthesis was stabilized by PMMA in another two cases. The other one case did not accept any operation.

About 11.11% (5/45) patients developed deep infection at a mean of 27.35 months (range from 1.6 to 47.6 months) postoperatively, including 2 patients with osteosarcoma in femur and 3 patients with osteosarcoma in tibia. Thus, the infection rate is 5.7% in femur and 30% in tibia. One infection occurred during the chemotherapy period, and four infections occurred outside the chemotherapy period, more than 6 months (range from 6.97 to 17.8 months) after operation procedure. All patients underwent operation for the infection including amputation in one case, washout and a 6-week course of antibiotics in one case, one-stage revision and re-implantation of the primary prosthesis after sterilization in one case, and two-stage revision with antibiotics-containing bone cement spacer replacement and implantation of new prosthesis after infection control in two cases (Fig. 6a–c). No patients have any evidence of re-infections until the last follow-up.

There were two cases of peripheral fracture of prosthesis, which were treated by open reduction and internal fixation with plate in one case and external fixation in another case with undisplaced fracture. Other complications include dislocation of knee joint in one case, tear of patellar tendon in one patient with osteosarcoma in tibia, and injury of nervous peroneus communis in two cases.

Twenty patients were evaluated functionally using the MSTS scoring system for limb salvage until the last follow-up, and the average MSTS score was 83.2% (24.95/30 on average, range from 70 to 100%). All patients with limb salvage could walk without support. About 85% of patients did not suffer pain from the affected limbs, and the average point is 4.8. Twenty percent of patients had normal gait, 30% of patients were in minor cosmetic grade, and about half of the patients oscillated between normal and minor cosmetic gait. The average point of gait was 3.9. As for walking component, 55% of patients could walk unlimitedly, 5% of patients with limited walking, and 40% of patients with slightly limited walking (grade 4). In case of function component, 55% of patients were recreationally restricted, 15% were not functionally restricted, and 25% were in between (grade 4). In the aspect of functional acceptance, 75% of patients were satisfied or more than satisfied and 25% could accept it.

## Discussions

The mortality and morbidity of bone sarcomas are high for children and young adults. About 66% of bone sarcoma occurred around the knee [8]. With the development in chemotherapy, diagnostic imaging methods, and surgical techniques, their long-term survival rates have increased from 60 to 70% [9]. Limb reconstructions maintaining maximal function for these young patients pose a serious challenge to the surgical team especially when the sarcoma involves the growth plate in the lower limb. Biological reconstructions such as rotationplasty, vascularized bone transfer with or without allografts, or osteoarticular allografts alone produce good function but are restricted with various disadvantages including unfavorable appearance, infection, fracture, and limb length discrepancy [10, 11]. Endoprosthetic replacement with extendible prosthesis has been adopted for skeletally immature patients with malignant tumor in the extremity, especially in the lower extremity since 1976. Some mid- and long-term results of the extendible prosthesis have been reported [4, 12] Encouraging functional result has been achieved even though with high complication rate. This study presents a relatively large cohort of patients with extendible prosthesis performed in the same center with the same surgical team. The prosthesis adopted in the current study is custom-made and economical, which could preserve as much bone as possible. The prosthesis is extended with relatively small incision, and the extension length is secured by the insertion of two half collars binding on the piston. Thus, mechanical failure for extension was not observed in this study. With this kind of prosthesis, only one patient had amputation owing to prosthesis-related complication and 97.06% patients survived with limb salvaged. The local recurrence rate is 6.7%, and the 5-year overall survival rate is 72.7%. Our current study achieves similar clinical outcome as the study by Dotan et al. [13].

Despite the great advantages of maintaining the limb length, a high number of complications could be expected with the use of extendible prosthesis. In addition to all the complications found in adults treated with an endoprosthesis, there are some pediatric-specific complications such as problems correlated with circumferential and axial growth of bone, repeated operations for elongation, and mechanical failure caused by elongation mechanism. In general, the complications can be classified as soft tissue failure, mechanical structure failure, infection, and aseptic loosening [13]. The mode of complications varied in different studies while mainly attributed to different types of prosthesis implanted. In Schinhan et al.’s study [14], in which three kinds of extending mechanism had been applied for different prosthesis systems in a span of 27 years, soft tissue failures (46%) was the most common complication among these 184 complications, followed by structural failures (28%), infections (17%), and aseptic loosening (8%). In the medium-term study of the Stanmore noninvasive extendible endoprosthesis, complications developed in 16 patients (29.1%), among which deep infection was the most common one (10.9%), followed by structure failure (5.5%), periprosthetic fracture (5.5%), and aseptic loosening (1.8%) [4]. The most prevalent complication in the current study is aseptic loosening, followed by deep infection. Aseptic loosening is the main concern for the mechanical failure; 15.6% (7/45) of survived patients had prosthesis loosening, which may be owing to the circumferential growth of the bone shaft and deficiency of bone growth between the interface of bone and the prosthesis. According to our result, risk of prosthesis loosening was much higher in children younger than 10 years old compared to those of older than 10 years old. The stem for the younger patients can be as thin as 10 mm on average, while the diameter of the medullary cavity of the bone shaft increases with age. So, the prosthesis would loose and sink. No osteointegration is the main deficiency of this prosthesis, and it may contribute to the loosening of the prothesis. The bone absorption was observed at most of the bone prosthesis junction, which may be owing to the deficiency of bone growth in the interface and blood supply destruction of the internal and external periosteum. The introduction of the hydroxyapatite collar has been reported to decrease the loosening of the prosthesis [15]. The prosthesis design of the fixed hinge knee adopted in the current study may increase the risk of loosening than the rotating hinge knee according to a previous study [15]. The straight stem design may focus the stress on the end of the prosthesis and increase the risk of puncture of the cortex of femur shaft, while the curve stem may fit in with the physiological curvature of femur shaft and disperse the stress.

Deep infection is one of most common complications, which may even increase with a mean of 1% per year and 2.7% with any operation throughout the life of the prosthesis according to a previous study [12]. This complication was more significant in young patients due to their basic immunocompromised status, multiple operative procedures, and relatively poor soft tissue covers. The infection rate of the current study is 11.11%. Except one infection which occurred in the time frame of chemotherapy, all the other four patients occurred within 1.5 years after operation, which were likely related to the multiple surgical procedures, including implantation, lengthening, and revision. Poor soft tissue covers usually occur in the tibia and lead to the high risk of infection as compared to the femur prosthesis (30% vs 5%). Medial gastrocnemius flaps were used in every case, yet infection rates were still high. The infection often followed the open surgical procedure, and decreased surgical exposure seems to decrease the risk of infection. Even though infection was still the most common complication for noninvasive extendible prostheses, the risk of infection has been reduced to 10.9% after a mean follow-up of 41.2 months (range of 22 to 104 months).

Limb length equality is the main factor affecting the function of the leg. Restoration of the limb length is one of the primary goals in immature patients with bone tumor. By lengthening the extendible prosthesis, most of patients in the current study could keep the leg equality and achieve good functional result. Sixty nine percentage (22/32) of the surviving patients underwent elongating procedure in the current study. The main factors for patients not extending the prosthesis are local recurrence, complications including loosening or infection, overestimation of the limb length discrepancy, and individual reason including economic problem.

Extendible prosthesis cannot overcome all LLD problems in the skeletally immature patients. Extending potential of the prosthesis is limited by the length of the bone resected. In children, especially younger than 10 years of age, LLD can be 10–20 cm at the time of skeletal maturity after the resection of the distal femur, which could hardly be satisfied by prosthesis replacement one time. While revision of extendible prosthesis is challenging considering the impairment to the retained bone shaft when removing the previous prosthesis, the risk of infection and soft tissue contracture followed the operation. So, it seems critical to protect the unaffected growth plate adjacent to the tumor considering the safety margins and the healthy growth plate on the other side of the articular when implanting the stem. In the current study, even with a thin stem, the growth plate of the proximal tibia had been inhibited for the patient with sarcoma in the distal femur. In the study of Cool et al., the mean proximal tibial growth in the affected leg was 69% of the unaffected tibia, and the mean distal tibial growth in the affected tibia was 96% of the unaffected tibia [7]. In the study of Arteau et al., 65% of the patients experienced less proximal tibial growth in the operated as compared with the contralateral limb [16]. The growth inhibition may be due to the destruction of the physical blood supply and the ring of LaCroix during the surgery, and the direct impairment of the growth plate when the intramedullary stem crosses the physis. In the current study, the bone growth in the tibial stem may further restrict the growth of proximal tibia. The polyethylene sleeve placed through the proximal tibial physis allows the tibial stem to slide within, and it may reduce the resistance of growth of proximal tibial physis. In addition, the insertion point of the tibial stem must be precise to avoid increasing the defect of the growth plate and the formation of bony bar which would prevent the growth. Preservation of the subchondral bone cortex when removing the cartilage of the tibia plateau and avoiding violent manipulation can prevent the compression fracture of the growth plate (Salter-Harris type V). Considering the growth retardation can reach as high as 4 cm especially in little children, the necessity of placement of the tibial fixation should be evaluated. Semi-joint extendible prosthesis could avoid interference of the proximal physis of tibia which might be an alternative temporary prosthesis for the children before growth peak [17]. However, it associated with increased risk of prosthesis-related complications such as dislocation and knee instability. If the physis is not invaded by the tumor as indicated by the MRI, physeal distraction could be performed which allows for preservation of the epiphysis and reduces LLD in the skeletally immature patients [18, 19]. Under the navigation with fused CT-MR images, computer-assisted precise planning and execution of surgery could resect the tumor with safe margins and preserve the joint [20]. Thus, the biomedical advance has been offering more alternative treatments of limb salvage for skeletally immature patient. Selection of surgical procedures should be determined individually, considering the chemotherapy response, diagnostic image information, and complete tumor resection for each patient.

## Conclusions

The custom-made extendible prosthesis offers limb salvage with satisfactory function, while aseptic loosening, infection, and LLDs are still its main complications. Improved prosthesis design and individualized choice of operation and reconstruction methods according to the patient and tumor conditions will help to decrease the risk of complications and achieve better outcome.

## Data Availability

The datasets are available from the corresponding author on reasonable request.

## Abbreviations

ADM: Adriamycin
AVI: Adriamycin+VP-16+ifosfamide
DDP: Cisplatin
HDMTX-CF: High dose methotrexate with citrovorum factor rescue
IFO: Ifosfamide
LLD: Limb length discrepancy
MSTS: Musculoskeletal Tumor Society
